# Automatic Segmentation of Plants and Weeds in Wide-Band Multispectral Imaging (WMI)

**DOI:** 10.3390/jimaging11030085

**Published:** 2025-03-18

**Authors:** Sovi Guillaume Sodjinou, Amadou Tidjani Sanda Mahama, Pierre Gouton

**Affiliations:** 1 ImViA, UFR Sciences et Techniques, Université de Bourgogne Europe, 21078 Dijon, France; guillaume-sovi_sodjinou@etu.u-bourgogne.fr; 2 Institute of Mathematics and Physical Sciences, University of Aboney Calavi, Dangbo 01 BP 613, Benin; amadou.sanda@imsp-uac.org

**Keywords:** agronomic images, automatic segmentation, CAVIAR dataset, multispectral images, U-net, Pif-net

## Abstract

Semantic segmentation in deep learning is a crucial area of research within computer vision, aimed at assigning specific labels to each pixel in an image. The segmentation of crops, plants, and weeds has significantly advanced the application of deep learning in precision agriculture, leading to the development of sophisticated architectures based on convolutional neural networks (CNNs). This study proposes a segmentation algorithm for identifying plants and weeds using broadband multispectral images. In the first part of this algorithm, we utilize the PIF-Net model for feature extraction and fusion. The resulting feature map is then employed to enhance an optimized U-Net model for semantic segmentation within a broadband system. Our investigation focuses specifically on scenes from the CAVIAR dataset of multispectral images. The proposed algorithm has enabled us to effectively capture complex details while regulating the learning process, achieving an impressive overall accuracy of 98.2%. The results demonstrate that our approach to semantic segmentation and the differentiation between plants and weeds yields accurate and compelling outcomes.

## 1. Introduction

Multispectral imaging is extensively used across various domains, including remote sensing [1], facial recognition [2], and precision agriculture [3], among others. Multispectral image acquisition systems are highly diverse, particularly those utilizing scanning mode, where images are captured frame by frame. These systems fall into three main categories: tunable filter cameras, tunable illumination cameras, and multi-camera systems. Tunable filters, such as the LCTF (Liquid Crystal Tunable Filter) [4] and AOTF (Acousto-Optical Tunable Filter) [5], employ electronic techniques to capture different spectral bands. While they generate fully defined multispectral images, their acquisition time is too long for real-time applications.

On the other hand, snapshot acquisition systems capture multispectral images in a single exposure. Among these, single-sensor multispectral systems are divided into multiple categories. A commonly used approach involves a one-shot camera with a single sensor, combined with a multispectral filter array (MSFA). This compact and cost-effective solution enables real-time image acquisition, capturing all required spectral bands simultaneously. Wideband multispectral imaging (WMI) is particularly effective for analyzing plant pigmentation characteristics [6,7]. Our initial research focused on color images, which are widely accessible and inexpensive. However, these images are limited to three spectral bands within the visible range, restricting their analytical capabilities. In contrast, multispectral images incorporate bands, providing essential information about the chlorophyll content of plants.

Despite its advantages, WMI faces certain challenges. The uneven distribution of plant species and the indistinct boundaries between them reduce the effectiveness of conventional segmentation methods. Additionally, WMI’s spectral insensitivity to dark plants creates ambiguities, making it more difficult for existing techniques to extract meaningful plant features.

The localization and classification of plants and weeds in multispectral images pose significant challenges in the field of agriculture [8]. While deep learning has been effectively applied to a wide range of object detection and semantic labeling tasks, semantic segmentation of crops in multispectral images still encounters specific limitations [9]. The main challenges stem from several factors: (1) the inherent characteristics of plants, (2) the scarcity of large-scale labeled datasets, which limits the generalization capabilities of existing solutions across different images, and (3) the morphological variations and appearances within the images [10].

In response to these challenges, previous research has investigated the use of spectral features to classify plant species. This has been achieved by combining deep convolutional neural networks with handcrafted feature detectors and support vector machines [11]. These methods have significantly enhanced the accuracy of plant identification. However, they depend on a rule-based integration of both low-level and high-level descriptors, which restricts their ability to capture the overall hierarchy of features [12].

A recent framework inspired by U-Net has been developed to directly predict specific categories of input features [13]. This model can utilize class information from the ground truth label map to generate point instance maps. However, it does not consider the relationships between point representations of features or the supervision of category-specific pathways, which limits its applicability.

In recent years, researchers have increasingly focused on the semantic segmentation of weeds and crops using agronomic images derived from color and multispectral imagery. This area of study has garnered considerable attention due to its potential applications in precision agriculture and the urgent need for effective weed control techniques. To address this challenge, various segmentation methods and deep learning models have been developed to improve the accuracy and efficiency of weed and crop segmentation.

Deep learning methods are notable for their unique architecture and remarkable capacity to learn rich, hierarchical, and discriminative features from raw color and multispectral images. Given the success of these models across multiple domains, they have found increasing application in precision agriculture for decision-making, particularly in tasks like weed and crop segmentation. For instance, ref. [14] introduced a new approach: a multi-task semantic segmentation method based on a convolutional neural network, which allows for the simultaneous detection of crops and weeds. Similarly, ref. [15] proposed a sequence of two deep learning networks for the classification of weeds and crops using RGB images. This approach utilizes an encoder–decoder segmentation architecture, which assigns each pixel to a class corresponding to either vegetation or soil.

Furthermore, ref. [16] developed a method for the automatic detection and assessment of weeds in agricultural fields. This method employs a tunable acousto-optic hyperspectral sensor along with a detection algorithm. Soil–crop segmentation was performed using two spectral channels selected from the 100 available channels of the hyperspectral sensor. The weed detection relies on texture features extracted from the segmented images. The algorithm was tested on a database of images capturing cotton plants and weeds in their early developmental stages. The results demonstrated strong detection capabilities, identifying weeds in all images; the area infested by weeds was estimated with an error margin of 14%, and the false detection rate was 15%.

In another study, ref. [17] utilized three broad bands for the automatic detection of weeds in corn fields, using plant pixels that included both crops and weeds. More recently, Wang, Kaixin, and colleagues ref. [18] have worked on weed detection and recognition in complex wheat fields based on an improved version of YOLOv7. Meanwhile, ref. [19] employed a U-Net-based encoder–decoder architecture that integrates ResNet50 as a feature extractor to effectively perform weed recognition in sugar beet fields.

This paper, which proposes an algorithm for the segmentation of plants and weeds in a wideband multispectral image, is organized as follows: Section 2 describes the materials and methods, Section 3 presents the results, and Section 4 discusses the results.

## 2. Materials and Methods

### 2.1. Images

**Figure 1:**
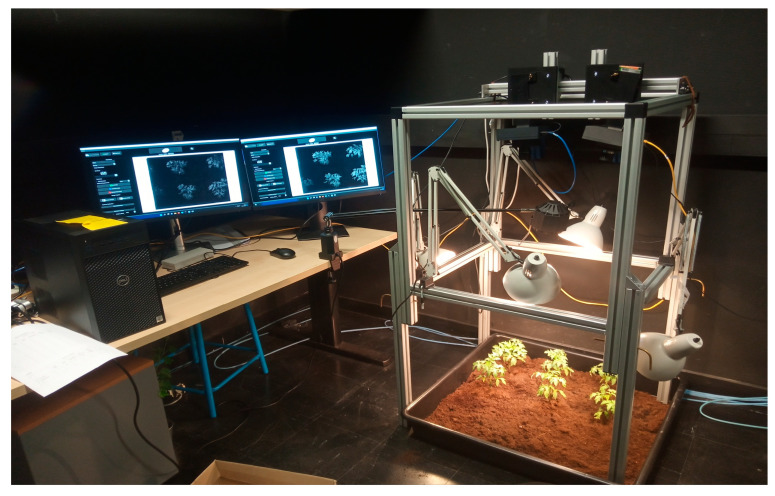
Set-up for MS image acquisition. (This camera was funded by the UE PENTA/CAVIAR project).

In this study, we utilized 500 broadband multispectral agronomic images from the CAVIAR database at the University of Burgundy [20]. These databases provide a diverse array of agronomic images, varying by lighting conditions and the presence of both plants and weeds. Two multispectral cameras were employed to capture images of the same scenes: one camera focused on the visible spectrum, while the other captured data in the near-infrared spectrum, as shown in Figure 1. We used both original mosaicked images and annotated images for our supervised techniques and MSFA (Multispectral Feature Analysis) technology.

The MSFA involves mosaics of filters arranged in a matrix, with each filter corresponding to a specific spectrum. In this study, we implemented a set of eight filters based on the Fabry–Perot principle, applied in the visible and near-infrared ranges [3].

**Figure 2:**
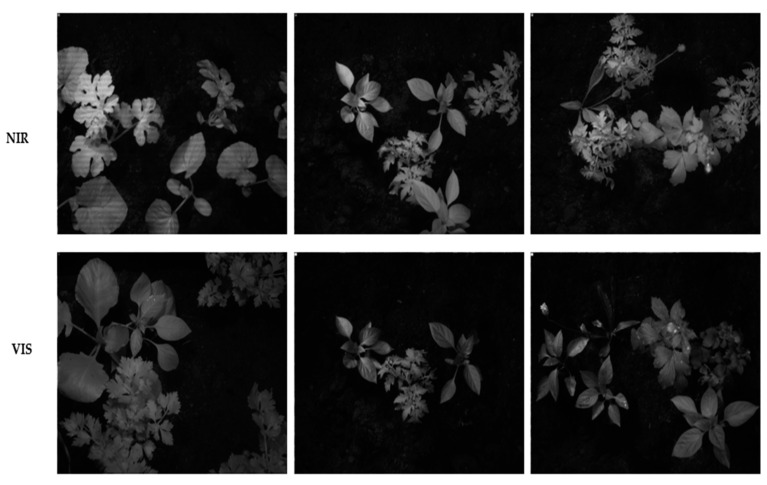
Example images taken by the cameras from different plants. The cameras were mounted beside each other, and the images of the scene were taken by the cameras simultaneously. (**Top row**): VIS image; (**bottom row**): NIR image.

Figure 2 shows several images captured by the cameras of different plants. VIS and NIR images were taken simultaneously for the same scenes. These raw images consist of eight single-band images, which will be obtained through demosaicking. The lighting used came from three halogen lamps, chosen to provide sufficient energy for both VIS and NIR.

Shrestha et al. [21] designed a multispectral (MS) camera by placing MS filters in front of the image sensors of a 3D camera equipped with two sensors. More recently, Zhang et al. [22] developed an MS camera for the visible spectrum, characterized by its compact design, portability, and high spatial resolution.

**Figure 3:**
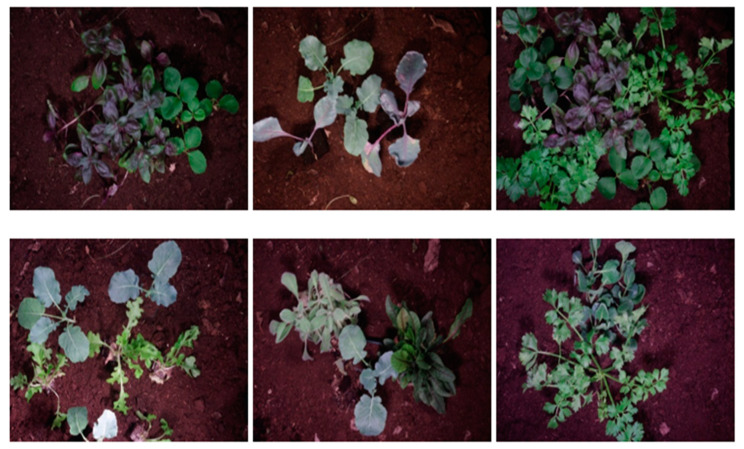
A color representation of the scene from which the MS images were obtained.

The plants were recently imported to the lab for imaging. They were positioned by their roots in the imaging setup, which helped maintain their health throughout the acquisition process, as shown in Figure 3.

### 2.2. Spectral Bands

The spectral bands used for MSFA creation were determined through simulations employing a genetic algorithm (GA). Table 1 outlines the details of the band selection simulations for the VIS and NIR ranges, achieving goodness of fitness coefficients (GFCs) of 99% and 98%, respectively, at a 99% probability level. The standard deviation was minimal, with RMS values of 0.012 for VIS and 0.011 for NIR. Consequently, sixteen optimized Gaussian filters were derived using the genetic algorithm, based on the Wiener fitness function.

**Figure 4:**
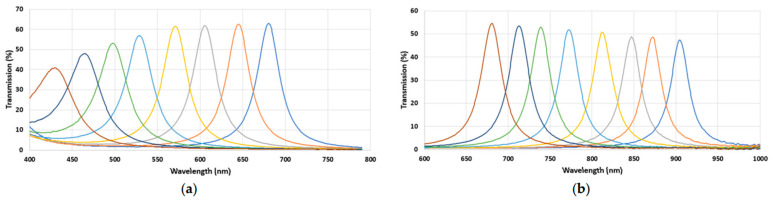
Spectral sensitivities of the MSFAs measured before the hybridation on the sensor; (**a**) VIS and (**b**) NIR. The lines represent the different spectral bands, and the colors have no particular significance. They were chosen randomly to visually distinguish each band.

Figure 4 illustrates the spectral sensitivities of the chosen filters after fabrication. This figure presents the relative sensitivities of each filter. It can be seen that the transmission rates of all bands were over 40%, and very good consistency was observed for the primary prototypes.

**Figure 5:**
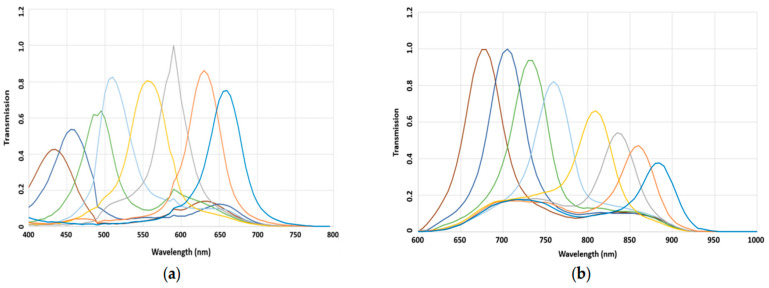
Spectral response of the cameras after hybridation process. (**a**) VIS hybrid sensor; (**b**) NIR hybrid sensor. The lines represent the different spectral bands, and the colors have no particular significance. They were chosen randomly to visually distinguish each band.

Figure 5 illustrates the spectral sensitivity of the MS image sensor. The lowest filter responses were found at 425 nm and 885 nm for the VIS and NIR MSFAs, respectively.

### 2.3. Refinement of Reference Images and Their Masks

**Figure 6:**
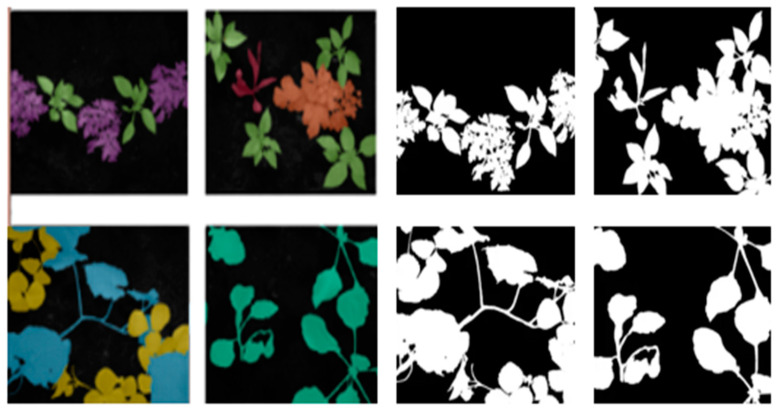
Examples of annotated ground truth images along with their corresponding masks.

To ensure an accurate evaluation of the segmentation model, we created ground truth masks by combining manual annotations with automated validation measures. This hybrid approach optimizes the reliability of the results and enhances the quality of the evaluation. Each plant is meticulously labeled with specific colors and annotations across all images. Figure 6 illustrates examples of these annotated images alongside the corresponding mask images, highlighting the accuracy of the annotations, especially along leaf edges and critical curves. These high-quality annotated images are essential for various segmentation tasks and deep learning techniques during both the training and testing phases of model development. By integrating these ground truth images into the training process, models can effectively learn to distinguish between different object classes, accurately estimate their positions, and recognize complex patterns, ultimately enhancing their performance and reliability.

The process starts by converting the image from the sRGB color space to the Lab color space, which significantly enhances segmentation capabilities. Next, the K-means clustering method is applied to the a and b components of the Lab color space. This method effectively groups pixels with similar chromatic characteristics into distinct clusters. These clusters are then used to assign labels to each pixel in the image, resulting in a segmentation that divides the image into homogeneous regions.

**Figure 7:**
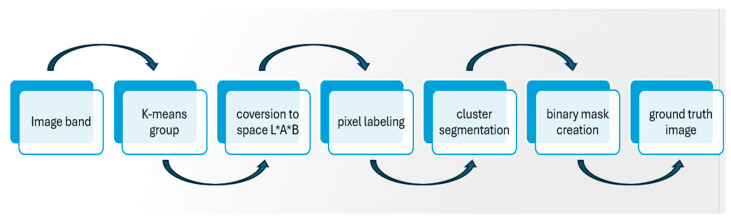
Block diagram of ground truth segmentation method.

Each region, defined by a specific cluster, is subsequently extracted to create individual segmented images, retaining only the pixels that belong to the same cluster. This meticulous segmentation process enables detailed analysis of various elements within the histological image, such as cells and tissue structures. Below, we outline the algorithm used to label the different images, detailing the steps taken to achieve this precise segmentation, as illustrated in Figure 7.

**Figure 8:**
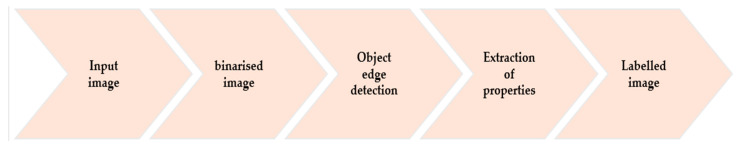
Block diagram of the image labeling method.

The diagram below illustrates the different steps of the image labeling process using MATLAB R2018a. From image acquisition to the display of identified objects, each phase contributes to effective segmentation and the assignment of a unique label to each detected region, as shown in Figure 8. This process is essential for analyzing and processing images in various applications, such as object recognition and image analysis in precision agriculture.

**Figure 9:**
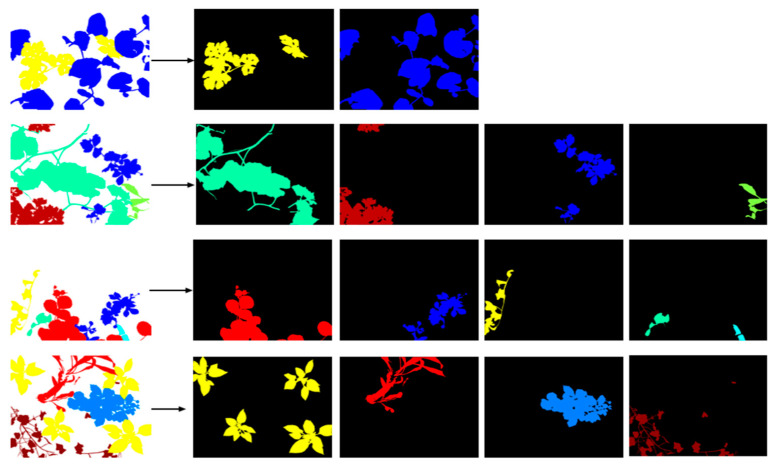
Examples of extraction of binary masks from labeled images.

In addition to the annotated images, we have provided binary masks in the database (Figure 9)

These masks are images of the same size as the original images and provide labels corresponding to only a single plant. These masks will help researchers more easily build their dataset for deep learning applications, as each plant can be introduced separately into the network.

### 2.4. Proposed PIF-Net and U-Net for Instance-Based Semantic Segmentation with Noise Reduction

Instance semantic segmentation is a technique that assigns a specific label to each pixel in an image while distinguishing individual objects within the same class. Unlike traditional semantic segmentation, which classifies each pixel with just one label without differentiating between individual instances, instance segmentation allows for the identification of separate objects that belong to the same class. The proposed approach yields pixel-level predictions [23], where each pixel is associated with a specific category as well as a unique instance.

In our work, we utilize a modified version of U-Net networks. In a traditional U-Net architecture, a series of convolutional layers is interspersed with max pooling layers that progressively reduce the resolution of the input image. This is followed by additional convolutional layers combined with upsampling operators that gradually increase the image resolution.

Our proposed method enhances this architecture by incorporating zero-padding in the convolutional layers, which ensures that the input and output dimensions remain consistent throughout the network. This modification not only preserves spatial information but also supports more effective feature extraction and reconstruction, ultimately improving the performance of the segmentation task.

To improve segmentation accuracy, we have integrated the PIF-Net (Part-based Instance Feature Network) into our workflow. The PIF-Net is specifically designed to extract detailed features at the object instance level, which allows for efficient fusion of these features before reconstruction [24]. This approach effectively preserves essential details for each object instance while reducing ambiguities between different instances of the same class.

After the features are extracted and merged by the PIF-Net, the resulting data are passed to the U-Net for semantic segmentation. The U-Net utilizes the high-level information previously gathered to ensure precise segmentation [25,26,27].

**Figure 10:**
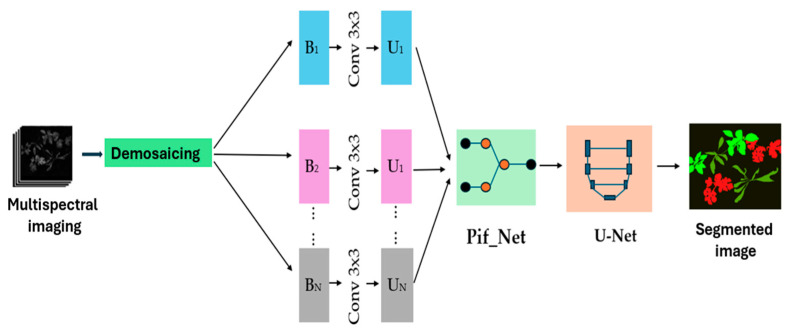
The diagram illustrates a CNN-based segmentation approach that uses the U-Net architecture in combination with PIF-Net.

Figure 10 illustrates the complete architecture of the network, along with the input and output images generated after the segmentation process. This integration of PIF-Net with U-Net not only improves the accuracy of segmentation but also enhances the overall quality of the results.

### 2.5. The Pif-Net Network

The proposed algorithm starts by demosaicking all mosaic images captured in the visible and near-infrared spectra. In this study, we introduce a two-stage framework for segmenting plants and weeds, utilizing PIF-Net and U-Net. The first stage of our approach employs a feature-level image fusion method inspired by [28]. This feature extraction module includes two subnetworks that share the same structure but have different weights. The upper subnetwork processes an eight-band multispectral (MS) image from the visible spectrum, while the lower subnetwork handles an eight-band multispectral image from the near-infrared (NIR) spectrum.

Both feature extraction subnetworks include three consecutive convolutional layers, each utilizing 3 × 3 convolutional kernels, followed by a Parametric Rectified Linear Unit (PReLU) [29,30]. We extract complementary information from the VIS and NIR multispectral images using two feature maps generated by the feature extraction module. To enhance agronomic insights, we fuse the extracted feature maps to create a multispectral image with high spatial and spectral resolution.

This fusion process enhances the information utilized, assigning weights to each channel. Initially, we concatenate the feature maps from both sets of complementary information in a linear manner. Subsequently, we apply an attention mechanism to derive a one-dimensional vector that represents the importance of each channel’s weight. This weight is then applied to the corresponding channels, allowing us to enhance valuable information while minimizing the impact of irrelevant data.

After performing feature fusion, the next step is to recover high-resolution multispectral images (HRMSI) from the combined features. To achieve this, we utilize three layers of 3 × 3 convolutional kernels to reconstruct the images from the fused features, allowing us to obtain the HRMSI across all eight bands.

The PIF-Net was trained using the dataset from our CAVIAR database, which consists of over 500 image volume cases across approximately 20 different modalities. Notably, this is the first known instance of utilizing a convolutional neural network (CNN) for broadband multispectral agronomic image fusion, as shown in the figure below.

**Figure 11:**
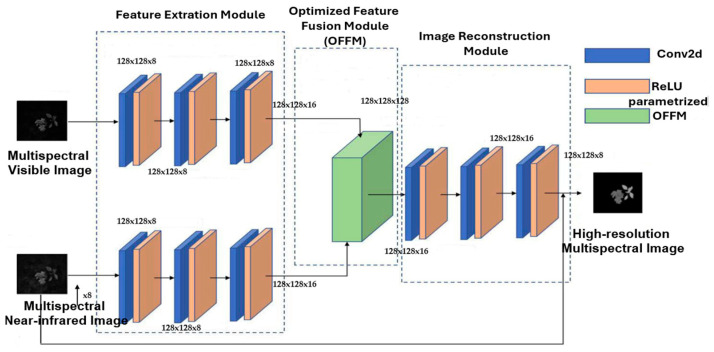
Overview of a dual-branch agronomic image fusion network using an attention mechanism to optimize feature fusion.

The architecture of PIF-Net features three distinct branches dedicated to feature extraction, feature optimization, and image reconstruction from the fused feature maps, as shown in Figure 11. This innovative design enhances the network’s ability to effectively process and integrate complex multispectral data, paving the way for improved analysis in agronomic applications. Figure 10 illustrates this process.

### 2.6. U-Net Proposed Architecture

**Figure 12:**
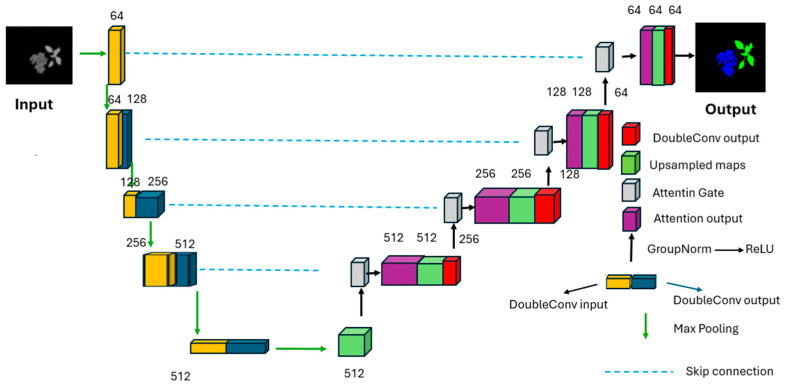
Illustration of the modified U-Net architecture.

In the second step of our proposed approach, we utilize the reconstructed and optimized images, which demonstrate extremely high multispectral resolution, for semantic segmentation. The selected fused images will be used as inputs for a semantic segmentation model, specifically U-Net, with zero-padding applied to ensure that the output images are the same dimensions as the input images [31]. This step enables us to obtain segmentation results, as illustrated in Figure 12.

## 3. Results

In this study, we utilize two different performance measures: the first is based on human visual perception, while the second is derived from specific mathematical calculations. Additionally, we evaluate the segmentation model using various metrics, including segmentation accuracy, Recall, Dice Coefficients (DSC), Mean IoU, the Mean BF Score and Root Mean Square Error (RMSE), which measures the deviation between predicted and actual values. This comprehensive evaluation approach provides a thorough analysis of the model’s performance.

### 3.1. Segmentation Evaluation Metrics

To evaluate segmentation accuracy, we use two commonly employed metrics: the Dice Coefficient and Intersection over Union (IoU). The Dice Coefficient, defined in Equation (2), measures the similarity between two data sets, typically comparing the model’s prediction with the ground truth. The IoU, defined in Equation (9), calculates the degree of overlap between the segmentation output and the ground truth. Together, these two metrics offer a robust and complementary evaluation of the segmentation model’s performance [32]. 

#### 3.1.1. Dice Loss

The Dice Loss function is a commonly used loss function in image segmentation tasks, particularly for semantic segmentation and instance segmentation. It is directly derived from the Dice Coefficient (Dice Similarity Coefficient, DSC), a metric that measures the similarity between two sets, typically a prediction and a ground truth. Even in its simplest formulation, the Dice Loss is somewhat adapted to handle class imbalance [33]. The Dice Loss is defined as follows:

(1) Dice Loss=1−Dice Coefficient, 

where the Dice Coefficient is given by:

(2) $$Dice=2\times \frac{\left|S⋂G\right|}{\left|S\right|+|G|},$$

In this context, *S∩G* represents the overlap between the ground truth and the segmentation result, while *S + G* denotes the total number of pixels in both *S* and *G* (their union). A higher Dice score indicates better segmentation quality. A lower Dice Loss indicates better segmentation performance, meaning that the predicted mask is very close to the ground truth mask.

The standard deviation of the Dice Coefficient is calculated in the same way as for any other numerical variable. The formula is:

(3) Diceδ=1N∑i=1NDicei−μ2

where:

*N* is the total number of samples,

${Dice}_{i}$ is the Dice Coefficient for sample i,

μ is the mean Dice score across all samples:

(4) $$\mu =\frac{1}{N}\;\sum_{i=1}^{N}{\;Dice}_{i}$$

#### 3.1.2. Mean IoU

Mean Intersection over Union (Mean IoU) is a widely used metric for evaluating the performance of image segmentation models. It measures the overlap between the predicted segmentation mask and the ground truth mask. IoU is calculated for each class by dividing the intersection of correctly classified pixels by the union of predicted and actual pixels. Then, the Mean IoU is obtained by averaging the IoU values across all classes.

A high Mean IoU indicates good segmentation, meaning the predicted region closely matches the actual region. This metric is robust against class imbalances and is extensively used in computer vision.

The Intersection over Union (IoU) for a given class is defined as:

(5) $$IoU=\frac{\left|A\cap B\right|}{\left|A\cup B\right|},$$

where:

A represents the pixels in the ground truth mask, B represents the pixels in the predicted mask, *A∩B* is the intersection of the two sets (correctly segmented pixels), *A∪B* is the union of the two sets.

The Mean IoU is then obtained by taking the average of the IoU over all classes *C*:

(6) $$Mean\;IoU=\frac{1}{C}\sum_{i=1}^{C}{IoU}_{i}$$

where *C* is the total number of classes.

This allows the algorithm to detect tree crown regions from terrestrial laser scans using an anchor-free deep learning model [34].

#### 3.1.3. Mean Boundary F1 Score

The Mean BF Score (Mean Boundary F1 Score) is a metric used to evaluate the accuracy of object boundaries in image segmentation tasks. Unlike Mean IoU, which assesses the overall overlap between the predicted and ground truth masks, the BF Score focuses specifically on the precision of detected edges. It is based on the F1 Score, which harmonizes precision and recall for boundary pixels. A high Mean *BF* Score indicates that the predicted object boundaries closely align with the actual ones.

The Boundary F1 Score (*BF* Score) for a given class is then calculated as:

(7) $$BF=1-\frac{2\times P\times R}{P+R},$$

where *P* is precision and *R* is recall.

The Mean *BF* Score is obtained by averaging the *BF* Score over all classes *C*:

(8) $$ean\;BF=\frac{1}{C}\sum_{i=1}^{C}{BF}_{i}$$

where *C* is the total number of classes.

A higher Mean *BF* Score indicates better boundary alignment between the predicted and actual segmentation masks. This metric measures the ability of the algorithm to distinguish between classes and is used to evaluate the overall performance of binary classifiers [35].

(9) $$IoU=\frac{TP}{TP+FP+FN},$$

Additionally, precision and recall are employed to evaluate the balance between false positives and false negatives. Both metrics are computed on a per pixel/voxel basis and are defined according to Equations (5) and (6), respectively.

(10) $$Precision=\frac{TP}{TP+FP},$$

(11) $$Recall=2\times \frac{TP}{TP+FN},$$

where *TP*, *FP*, and *FN* represent true positives, false positives, and false negatives, respectively.

Another measure is the “MRSE” coefficient, which determines the degree of similarity between the original segmentation and the resulting segmentation, as shown in Equation (10) [36]

(12) $$RMSE=\sqrt{\frac{1}{n}\sum_{i=1}^{n}{\left(y_{i}-{\hat{y}}_{i}\right)}^{2}},$$

${\left(y_{i}-{\hat{y}}_{i}\right)}^{2}$, measure the square deviation between reality and prediction for each individual point, which is an important basis for evaluating the overall performance of a statistical or prediction model.

### 3.2. Instance Segmentation Results Using PCA Combined to U-Net

**Figure 13:**
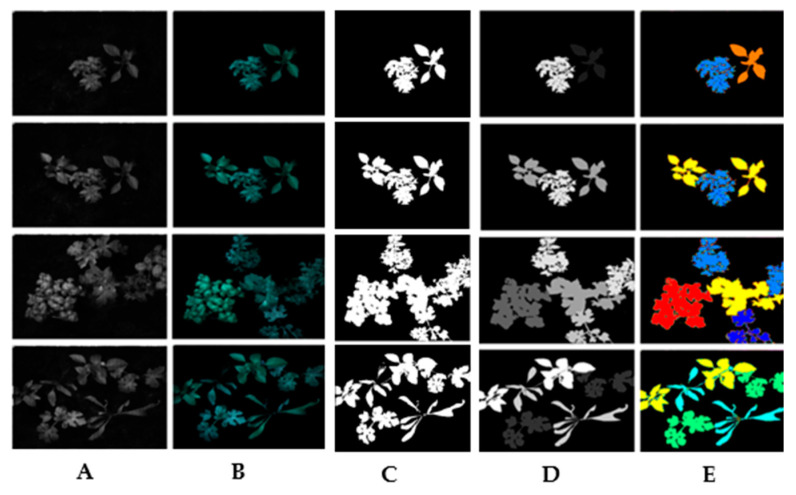
(**A**) Raw image: (**B**) 3-band image generated using PCA, (**C**) binary image mask indicating the valid segmentation region, (**D**) segmented image, (**E**) instance-segmented image.

In this experiment, Principal Component Analysis (PCA) is used to reduce the multispectral images to three bands and the different results are presented in Figure 13, after which the algorithm referenced in [37] is applied. During the training phase, the original dataset was divided into training, validation, and test subsets, comprising 70%, 15%, and 15% of the total data, respectively. After the validation set was made available, the networks were retrained using 85% of the training set to increase the number of examples, with the remaining 15% serving as the validation set.

### 3.3. Broadband Instance Segmentation

The PIF-Net network introduces an innovative codex–decoder architecture. The codex consists of two autonomous branches that incorporate Res-Pooling blocks and point attention blocks to efficiently extract features from the bands. The decoder, in turn, oversamples these features to enhance resolution. A hierarchical fusion module is integrated to enable adaptive fusion of band features at different levels, ensuring a complete and coherent integration.

This process generates combined features in both point and pixel representations, which are then fed into a classification module. This module is designed to perform multiple classification tasks and produce semantic segmentation results for both images and point clouds. For processing these images, a variant of the U-Net network is employed. In this variant, the initial set of convolutional layers is optimized to include the maximum number of pooling layers, gradually reducing the resolution of the input image. These layers are followed by additional convolutional layers, separated by upsampling operators that restore the image resolution. A key feature of this method is the use of “zero-padding” in the convolutions, which allows for the input and output images to maintain the same size. The final result of the semantic segmentation is a highly accurate and well-mapped image. The results obtained demonstrate the effectiveness of our multispectral image segmentation method.

To evaluate the accuracy and performance of the adapted U-Net model, both quantitative and qualitative measures were used. The evaluation methods are explained first, followed by a detailed presentation of the results. Analyzing these results provides insights into the performance of our method and its potential impact in the field of agronomy. Additionally, this method offers a broader framework for predictions, facilitating the training of convolutional neural networks (CNNs) with multi-resolution images. The networks were trained for 300 iterations using batches of 128 × 128 × 8, with an initial learning rate of 0.001 and a batch size of 10.

The algorithm that utilizes eight bands has demonstrated impressive effectiveness, achieving an accuracy of 98.2% in distinguishing plants from weeds. This underscores the potential of technology in precision agriculture. Additionally, the use of PIF-Net to enhance image quality by combining features from different bands represents a significant advancement. This approach not only leads to more accurate results but also streamlines the process of detecting and separating plants from weeds. With 500 multispectral images at your disposal, you have a strong foundation for obtaining meaningful results.

**Figure 14:**
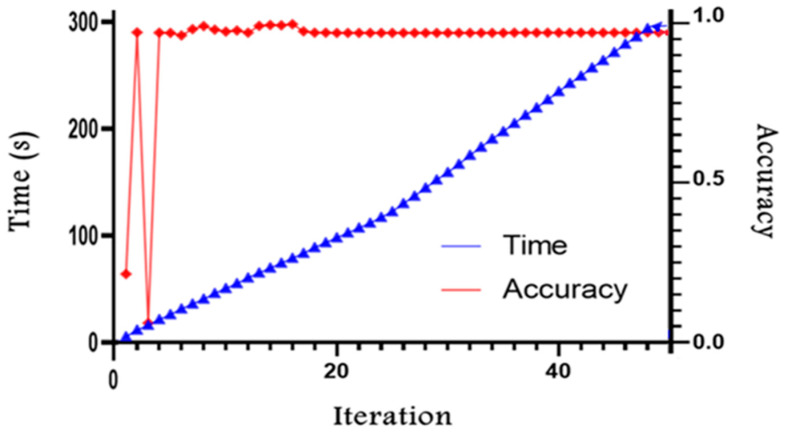
Changes in time and accuracy for different training iterations.

Figure 14 illustrates the evolution of training time and network accuracy as a function of the number of iterations. The blue curve shows a notable increase in training time, exceeding two minutes from the twentieth epoch onward. Each additional iteration thus significantly increases the overall computational cost. Meanwhile, the red curve reveals that accuracy fluctuates at first, then stabilizes around 0.98 starting at the twentieth epoch, indicating that the model reaches a performance level close to convergence.

A combined analysis of these two curves highlights that beyond the twentieth epoch, continuing the training does not yield any substantial gains in accuracy, while markedly increasing the total training duration. This observation underscores the need to strike a balance between performance and the time cost of training. In agricultural applications, where dozens of images are collected and need to be processed rapidly [38], this trade-off becomes particularly critical.

**Figure 15:**
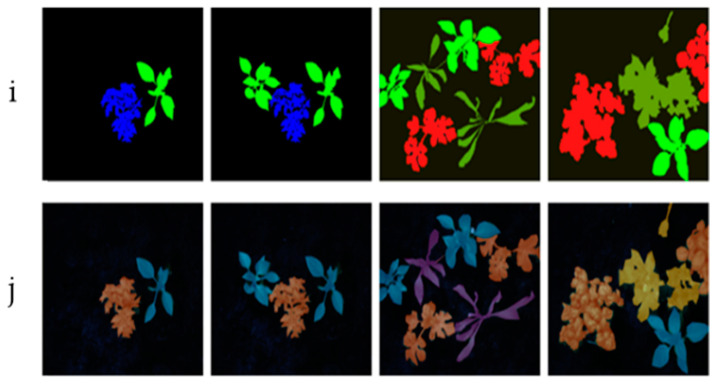
Semantic instance segmentation.

Figure 15 illustrates the results, showing that our feature fusion-based model achieves superior segmentation accuracy compared to other methods. This improvement is especially notable in accurately defining the edges of plants and weeds when compared to the ground truth reference images. The reference images are labeled and serve as benchmark data for segmentation, assigning each pixel to a specific class. They are essential for validating the segmented images using evaluation metrics.

In this figure, row (i) displays the segmented images generated by our M1 method after optimized feature extraction and fusion, while row (j) presents the corresponding ground truth images.

To evaluate the performance of our segmentation method, we utilize the Dice and Mean Root Square Error (MRSE) metrics. These indicators were selected for their ability to provide a comprehensive and objective assessment of segmentation quality. With these criteria, we can accurately measure the effectiveness of our approach and identify areas for improvement. For a fair assessment, we focus exclusively on the results from a single model, highlighting the best performances in bold and colored green for enhanced readability. Notably, the application of Pif-net for segmenting broadband multispectral instances demonstrates exceptional performance. This method achieves average Dice scores of 0.8281, 0.8279, 0.8235, 0.8233, 0.8261, and 0.8249 across three validation sets, along with MRSE scores of 0.0008, 0.0007, 0.0029, 0.0022, 0.0017, and 0.0011.

**Figure 16:**
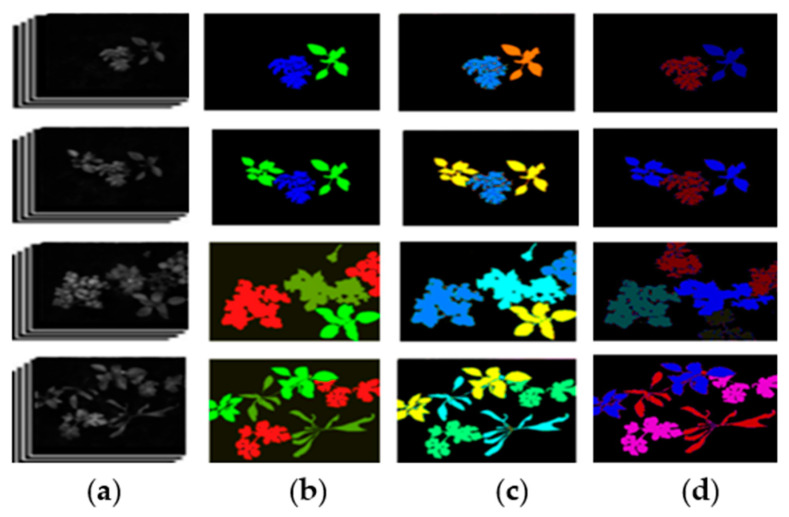
(**a**) multispectral image, Semantic segmentation of multispectral instances (**b**) Pif-net and U-net, (**c**) PCA and U-net, (**M3** (**d**)) utilizing intensity normalization on images.

While the performance of our method is slightly lower than that of some current state-of-the-art technologies, our primary goal is to showcase the effectiveness of pixel- and feature-level image fusion techniques for segmenting plant and weed instances. To highlight the efficacy of our approach, our results are compared against images segmented using the PCA method for three-band images, as well as against reference multispectral images segmented solely in the near-infrared using U-net, as shown in Figure 16.

This study examines the effects of the Pif-net module and U-net. It specifically focuses on the edge effects of plants and weeds in segmented images. Furthermore, the individual impacts of using Principal Component Analysis (PCA) and U-net are investigated, analyzing their effects in isolation.

After analyzing the three methods, we observe that the method in Figure 16b, which includes extraction, optimized feature fusion, and image reconstruction, shows a clear trend: segmentation errors are more frequent in the methods in Figure 16c,d, particularly in the blurred frontal areas between plant leaves, weed leaves, and soil. When comparing the three segmentation methods, we find that the method in Figure 16c struggles to accurately define the contours of plants due to a loss of information resulting from the exclusion of five other bands. In contrast, the method in Figure 16d encounters fewer difficulties, although it still shows some irregularities in contour definition.

In contrast, the first method, whose results are shown in Figure 16b, which likely incorporates techniques such as Pif-net, U-net, and 16 multispectral bands following image reconstruction, enables much more precise segmentation of plants and weeds, with clearly defined contours. This analysis underscores the importance of integrating multispectral information to enhance the quality of segmentation in image processing applications.

## 4. Discussion

This study provides valuable insights into the performance of different segmentation techniques. To conduct this research, we evaluated three distinct methods under various configurations, reporting key metrics such as Precision, Recall, Mean IoU, and Mean BF Score. These results are detailed in the tables below.

The results from Table 2 and Table 3 confirm that M1 stands out as the best segmentation method. It is distinguished by high precision, strong stability, and a minimal error rate.

Method M1: With an accuracy of 98.2%, M1 outperforms the other methods, delivering exceptionally reliable segmentation. This makes it particularly useful for tasks requiring precise detection, such as plant boundary identification in precision agriculture. The high accuracy of M1 suggests superior generalization across the test images, making it the most dependable choice for real-world applications. Dice Coefficient, RMSE error, recall, Mean IoU and mean BF score confirm the good performance of the method M1.

Methods M2 and M3: Although close in performance (97.1% and 97.2%, respectively), these methods show slightly lower effectiveness compared to M1. In scenarios where high precision is essential, these small differences in performance could have a significant impact on the results, making them less reliable than M1.

✓ M2 exhibits high variability, which could compromise its reliability in certain configurations.
✓ M3 is the least efficient, characterized by lower accuracy, reduced precision, and greater errors. The other metrics values are also bad.

Thus, this study highlights the clear advantage of M1 as the most effective and reliable segmentation method among those evaluated.

Table 4 presents the average performance values of the network for detecting weed coverage. The highest accuracies were achieved by training the network for more than 40 epochs. However, in all cases, the network’s accuracy exceeded 97% after just 20 epochs. When training was limited to 10 epochs for quicker results, the accuracy was still acceptable in most instances. In a related study, Giakoumoglou, Nikolaos, et al. [39] employed a method for the early detection of Botrytis cinerea symptoms using deep learning and multispectral image segmentation, achieving a high overall accuracy of 90.1%. Additionally, Gupta, Sanjay Kumar, et al. [40] developed an automated approach for precision agriculture that identifies multiple weed classes through semantic segmentation. Among the models evaluated, the U-Net-based Inception-ReseNetV2 model achieved the highest F1 Score, reaching 96.78%.

**Figure 17:**
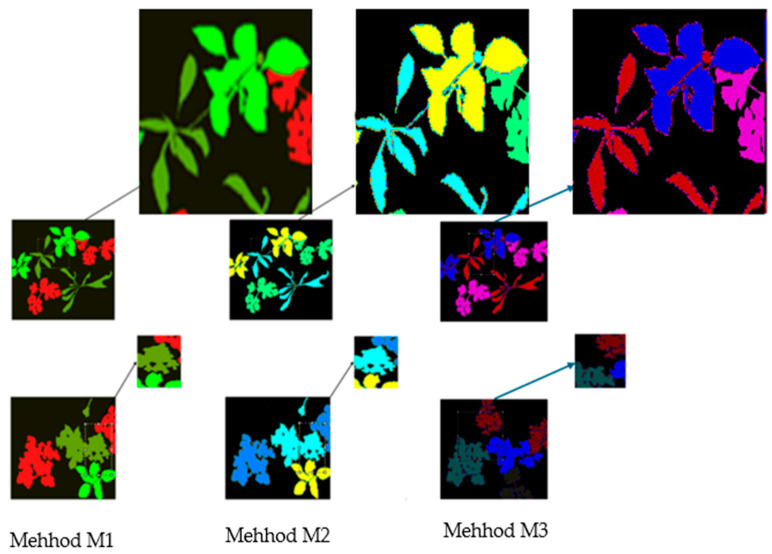
Observation of plant and weed borders using three different segmentation methods.

Based on the experimental results presented in Figure 14 and Figure 17, as well as Table 2, Table 3 and Table 4, we can make an additional observation: The M1 method, which integrates Pif-net and U-net, outperforms the M2 and M3 methods. This advantage is due to Pif-net’s ability to extract relevant pixel-level features for plants and weeds. This capability allows the model to focus on the most significant regions of the image, particularly the edges while enabling the network to capture important spatial contextual information crucial for effective segmentation.

Furthermore, the performance analysis of the M2 and M3 methods indicates that the combination of Pif-net and U-net notably improves segmentation accuracy. The integration of these two modules enhances the network’s ability to understand complex visual patterns associated with plants and weeds, resulting in overall superior performance.

## 5. Conclusions

In this work, we highlight innovative image fusion strategies applied at both the pixel and feature levels to improve the segmentation of plants and weeds. Our goal is to fully leverage the agronomic information contained in the multispectral bands for a more comprehensive and precise analysis of these instances. We have adopted a dual approach, extracting complementary information from the original image using two subnetworks. Each subnetwork has an identical architecture but variable weights. This configuration enhances the specific characteristics of each type of information, enabling us to effectively distinguish between plants and weeds. Our proposed algorithm demonstrates good performance, achieving a global accuracy of 98.2% in segmentation and efficiently separating plants from weeds. The framework we have developed is highly adaptable and can easily be tailored to accommodate other crops and types of weeds by fine-tuning the model with samples from these different crops. We believe that this adaptability noticeably enhances the framework’s versatility and utility, making it a robust solution for the identification and characterization of crops and weeds in precision agriculture.

In future works, we plan to use attention mechanisms to possibly improve performance. Other improvements could be achieved by optimizing hyperparameters and increasing training data to further refine segmentation performance.

## Figures and Tables

**Table 1:** Simulation results of band selection using GA.

Parameter	VIS	NIR
Min Delta E 2000	0.0047	0.0051
Max Delta E 2000	0.3500	2.2575
Mean Delta E 2000	0.0562	0.0937
Median E 2000	0.0465	0.0658
STD Delta E 2000	0.0402	0.1320
Min RMS	0.0020	0.0021
Max RMS	0.0740	0.0986
Mean RMS	0.0123	0.0108
GFC > 0.99	99%	98%
GFC > 0.95	100%	100%

**Table 2:** Network accuracy for different methods.

Criteria	Method M1	Method M3	Method M2
Accuracy	98.2%	97.2%	97.1%
Dice Coefficient	0.8279 ± 0.16%	0.8249 ± 5.17%	0.8233 ± 3.19%
RMSE	0.0007	0.0011	0.0022

**Table 3:** Global Method Comparison.

Criteria	M1	M3	M2
Accuracy	98.2%	97.2%	97.1%
Recall	0.89017	0.82643	0.78801
Mean IoU	0.92011	0.89711	0.88011
Mean BF Score	0.71082	0.48082	0.40082

**Table 4:** The average values for the accuracy of the network for different numbers of epochs.

Epochs	Time Elapsed (s)	Mini-Batch Accuracy	Mini-Batch Loss	Base Learning Rate
1	6	55.52	6.45	0.001
10	61	96.91	0.65	0.001
20	121	97.66	0.32	0.001
30	182	97.97	0.19	0.001
40	246	99.45	0.12	0.001
50	300	99.49	0.10	0.001

## Data Availability

Data are contained within the article.

## Abbreviations

The following abbreviations are used in this manuscript:

WMI	Wideband multispectral imaging
LCTF	Liquid Crystal Tunable Filter
AOTF	Acousto-Optical Tunable Filter
CNNs	convolutional neural networks
NIR	near-infrared
MS	multispectral
VIS	visible
PCA	Principal Component Analysis
PIF-Net	Part-based Instance Feature Network
PReLU	Parametric Rectified Linear Unit
HRMSI	high-resolution multispectral images
MSFA	multi-spectral filter arrays
RMSE	Root Mean Square Error
sRGB	standard RGB
